# Construction of Graphene/Fe_3_O_4_@Hollow Glass Microsphere Composite Foam with Excellent Electromagnetic Interference Shielding, Joule Heating, and Flame-Retardant Properties

**DOI:** 10.3390/molecules31162824

**Published:** 2026-08-13

**Authors:** Huan Yue, Shigang Li, Yixian Lv, Xueqing Wang, Jinlong Pan, Hao Wu, Heng Zhang, Hexin Zhang

**Affiliations:** 1 School of Chemistry and Chemical Engineering, Pingdingshan University, Pingdingshan 467000, China; 231005064@e.pdsu.edu.cn (Y.L.); 241005026@e.pdsu.edu.cn (X.W.); 251005009@e.pdsu.edu.cn (J.P.); 221027147@e.pdsu.edu.cn (H.W.); 241017069@e.pdsu.edu.cn (H.Z.); 2 School of Chemistry and Chemical Engineering, Anhui University of Technology, Ma’anshan 243032, China; 2002421lsg@ahut.edu.cn

**Keywords:** graphene, hollow glass microspheres, multifunctional, lightweight, flame-retardant

## Abstract

The development of lightweight multifunctional materials integrating electromagnetic interference (EMI) shielding, Joule heating and flame retardancy is highly demanded for advanced electronics and aerospace systems. Herein, we fabricate graphene/Fe_3_O_4_@hollow glass microsphere (G/Fe_3_O_4_@HGM) composite foam with an ultralow density of 0.36 g/cm^−3^. The porous structure synergizes graphene’s conductivity, Fe_3_O_4_’s magnetism and HGM’s low thermal conductivity to optimize impedance matching. The foam delivers absorption-dominated EMI shielding with a maximum X-band shielding effectiveness (SE) of 60.1 dB and an average absorption coefficient of 0.56, which effectively suppresses secondary electromagnetic reflection pollution. The composite exhibits stable voltage-controllable Joule heating: the 25 wt% Fe_3_O_4_@HGM sample reaches 91.3 °C at 16 V, enabling rapid de-icing within 200 s and stable thermal maintenance at −20 °C. Flame tests confirm no combustion or structural collapse under open flame. This work provides a simple fabrication strategy for lightweight multifunctional materials applicable to aerospace stealth, electronic thermal management and anti-icing systems.

## 1. Introduction

Fast development of electronics, aerospace and military equipment improves application efficiency yet causes severe electromagnetic pollution. Pollution of this type compromises the performance of nearby equipment and endangers human wellbeing [1,2,3]. Thus, the development of EMI shielding materials has become a key research priority, with particular attention on multifunctional non-metallic matrix composites that integrate multiple performance requirements [4,5,6,7,8].

Within the aerospace and military sectors, the accelerated evolution of infrared detection techniques has set higher standards for the infrared stealth properties of equipment systems [9,10,11]. Materials with excellent infrared stealth adjust emissivity and thermal conductivity to suppress target infrared signals and avoid detection from enemy infrared sensors [12,13,14]. However, traditional shielding materials face obvious drawbacks: metallic shields are dense and lack infrared stealth capacity; ceramic infrared stealth materials are non-conductive and brittle and unable to realize electrothermal heating or EMI shielding. This single-function bottleneck drives the development of composites integrating EMI shielding, Joule heating, infrared insulation and flame retardancy [15,16,17,18,19,20].

Graphene, a two-dimensional carbon nanomaterial with a unique honeycomb structure, has attracted extensive attention in the domain of functional materials by virtue of its excellent electrical conductivity [21,22,23]. As a case in point, Wei et al. [24] fabricated a GNS/PTFE composite thin film with multifunctional characteristics and a “brick-wall” microstructural morphology via repeated rolling. This film showed an EMI shielding effectiveness hitting 50.85 dB at its maximum and a reflection coefficient (R) of 0.91, together with prominent Joule heating performance and photothermal conversion efficiency. However, graphene’s high electrical conductance causes impedance mismatch with ambient air, leading to intense EM wave reflection at its surface and bringing about significant secondary electromagnetic pollution [25,26,27].

In recent years, composite foam-based materials have turned into a key research topic in multifunctional materials owing to their porous structure [28]. The interconnected porous network not only decreases the density of the material but also provides passages for multiple reflection and absorption of EM waves, thereby improving EMI shielding effectiveness. Simultaneously, the air retained in the pores substantially decreases the thermal conductivity of the material, a characteristic that supports infrared stealth [29,30,31]. Hollow glass microspheres (HGMs), serving as lightweight inorganic additives with a hollow spherical configuration, exhibit merits such as low density, low thermal conductivity, and excellent chemical stability. Their hollow structure reduces the overall density of the composite and creates a “confined space” that hinders heat transfer and EM wave propagation. Moreover, the smooth spherical surface of HGM acts as a dispersion carrier for graphene, effectively mitigating graphene agglomeration by preventing direct contact between graphene sheets. The synergistic effect between graphene and HGM provides a feasible strategy for preparing multifunctional composites. Additionally, binary graphene/HGM materials lack magnetic loss components, limiting microwave absorption and shielding efficiency. Pure graphene causes strong wave reflection due to unmatched impedance. Fe_3_O_4_ magnetic nanoparticles loaded on HGM introduce magnetic loss centers and abundant heterogeneous dielectric interfaces, balancing dielectric–magnetic dual loss to optimize air–material impedance matching. The HGM hollow skeleton further cuts density and prolongs microwave propagation paths.

Therefore, this work aims to fabricate lightweight G/Fe_3_O_4_@HGM composite foams and reveal the structure–property relationships between filler loading and multi-functional performance. We hypothesize that Fe_3_O_4_ magnetic coating can tune composite electromagnetic loss to shift shielding from reflection to absorption, while porous HGM reduces density and thermal conductivity for infrared stealth and thermal management. We systematically characterize the morphology, electromagnetic, electrothermal and flame-retardant behaviors to clarify internal synergistic mechanisms and offer a scalable preparation route for next-generation lightweight functional devices.

## 2. Experimental

### 2.1. Materials

Hollow glass microspheres (HGMs, trade name: K1, density: 0.125 g/mL) were provided by 3M China Ltd. (Shanghai, China). Ferric chloride hexahydrate (FeCl_3_·6H_2_O, ≥99%), ferrous chloride tetrahydrate (FeCl_2_·4H_2_O, ≥99%), ammonia solution (NH_3_·H_2_O, 25%), and sodium hydroxide (NaOH, ≥99%) were purchased from Shanghai Titan Scientific Co., Ltd. (Shanghai, China). Graphene was obtained from Maanshan Wanene New Materials Co., Ltd. (Maanshan, China). Silicone resin emulsion (SILRES^®^ MP 50 E) was supplied by Wacker Chemicals (China) Co., Ltd. (Shanghai, China). Dispersant (BYK 190) was purchased from BYK Company (Wesel, Germany).

### 2.2. Preparation of Graphene Conductive Ink

A total of 250 g deionized water, 350 g silicone resin emulsion, and 1 g dispersant were added to a 1000 mL jacketed stainless-steel reactor under continuous mechanical stirring. Subsequently, 100 g graphene powder was gradually added in batches of ~2 g to realize pre-dispersion. After complete feeding, the mixture was stirred at 800 rpm for 1 h to homogenize the graphene suspension. The mixed suspension was then transferred to a sand mill disperser for 2 h of high-speed grinding to fully exfoliate and disperse the graphene sheets, yielding homogeneous graphene conductive ink.

### 2.3. Preparation of Fe_3_O_4_@HGM

A total of 20 g sodium hydroxide (NaOH) was dissolved in 500 mL deionized water in a beaker. Subsequently, 15 g hollow glass microspheres (HGMs) were gradually introduced into the beaker, and the mixture was subjected to mechanical stirring at 500 rpm for 24 h. The treated HGM was filtered and rinsed multiple times with deionized water until the pH value reached 7. The HGM was subsequently redispersed in 300 mL deionized water. Under mechanical stirring, 200 mL deionized water containing 15.0 g FeCl_2_·4H_2_O and 40.5 g FeCl_3_·6H_2_O (pre-dissolved) was fed into the system. The system temperature was increased to 65 °C, and 120 mL ammonia solution (NH_3_·H_2_O) was introduced dropwise. The reaction continued under these conditions for 8 h. After the reaction, stirring was halted, and the mixture was allowed to settle undisturbed for 30 min. The upper floating material was harvested via suction filtration and rinsed multiple times with deionized water until the pH of the filtrate reached 7. The obtained Fe_3_O_4_@HGM was dried in a drying oven at 110 °C for 24 h.

### 2.4. Preparation of G/Fe_3_O_4_@HGM

**Scheme 1:**
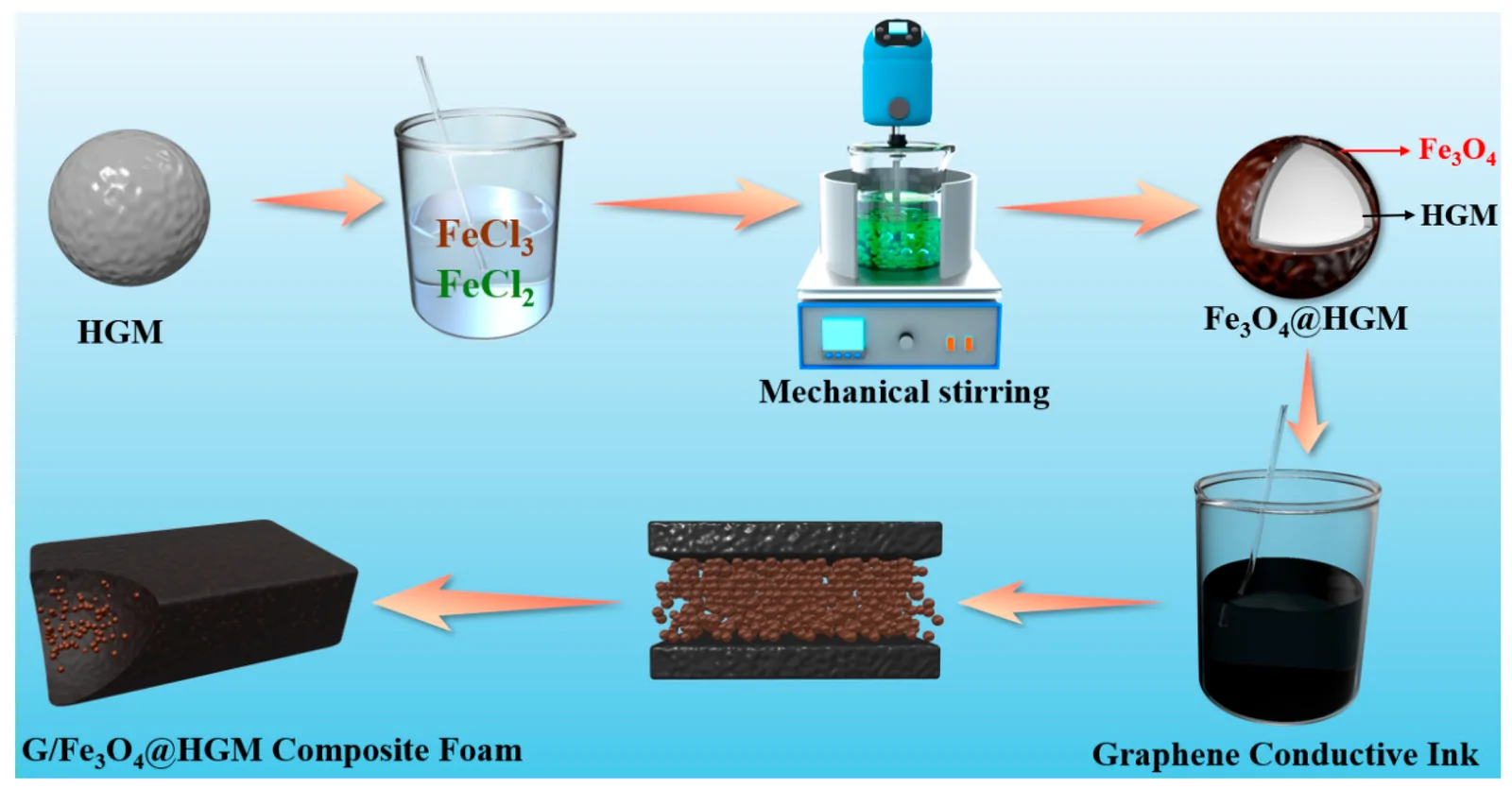
Schematic diagram of the preparation of G/Fe_3_O_4_@HGM composite foam.

As shown in Scheme 1, a predetermined amount of Fe_3_O_4_@HGM was added to 50 g graphene conductive ink. The mass ratios of Fe_3_O_4_@HGM filler relative to graphene ink were set as 25 wt%, 35 wt% and 45 wt%, respectively. The mixture was mechanically blended at 600 rpm for 30 min at room temperature to form a uniform mud-like G/Fe_3_O_4_@HGM composite slurry. The homogeneous slurry was transferred to an adjustable-thickness polytetrafluoroethylene (PTFE) mold and placed in a forced-air circulation drying oven under ambient air atmosphere at 70 °C. Drying lasted until the mass change in each sample was less than 0.001 g within 1 h to guarantee complete solvent evaporation. We prepared five parallel foams for each filler loading and ran all morphology, electromagnetic, electrothermal and flame retardancy tests to confirm repeatability.

The “characterization” section is provided in the Supporting Information.

## 3. Results and Discussion

**Figure 1:**
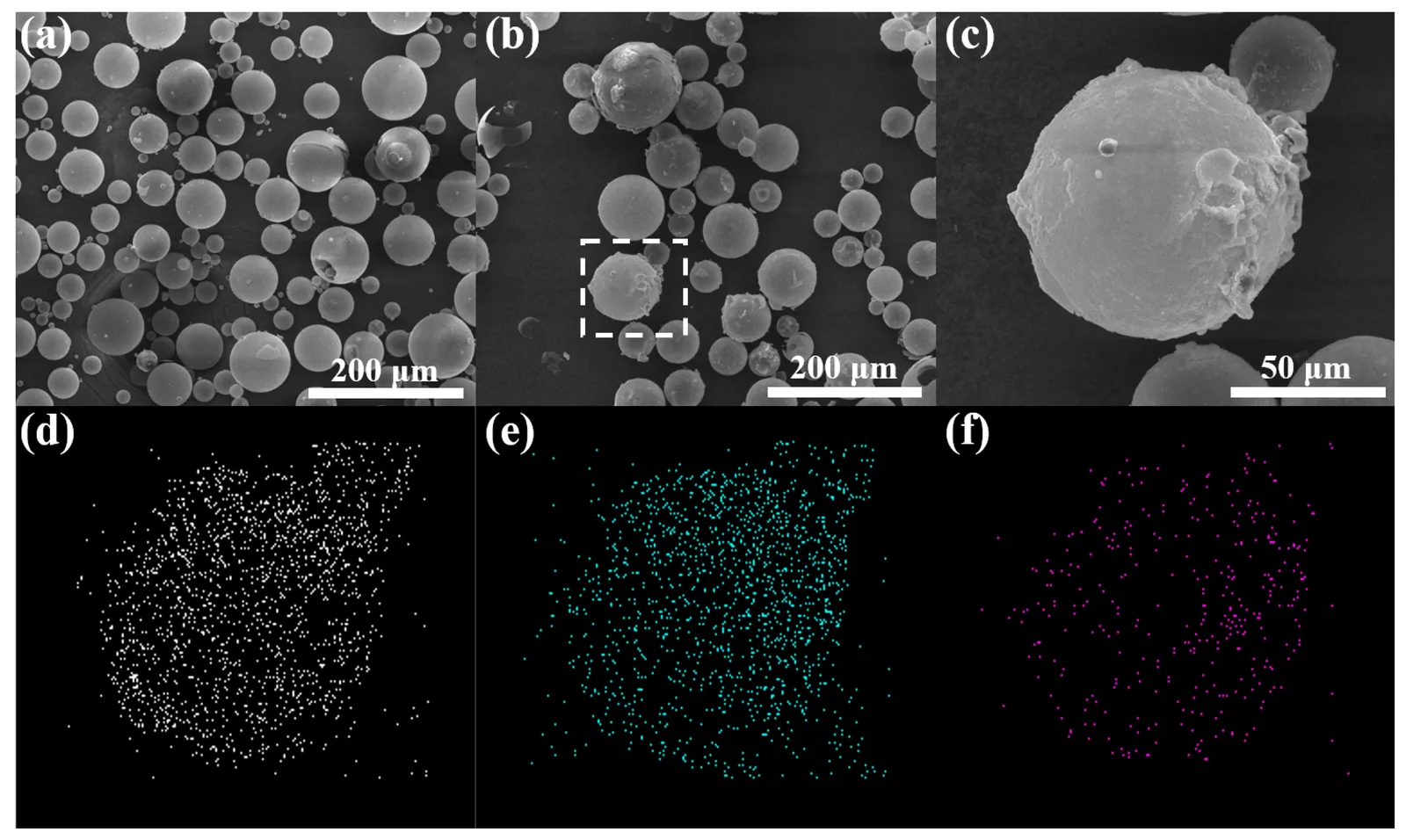
Morphology of HGM and Fe_3_O_4_@HGM. SEM image of (**a**) HGM; (**b**,**c**) Fe_3_O_4_@HGM; (**d**–**f**) elemental distribution mapping of (**c**).

Figure 1a displays the scanning electron microscope (SEM) micrograph of pristine hollow glass microspheres (HGMs). The surface of the original HGM appears smooth. Figure 1b,c are SEM images of Fe_3_O_4_@HGM; after Fe_3_O_4_ loading, the surface becomes significantly rough. Figure 1d–f shows the elemental mapping of the area in Figure 1c. The main elements are silicon (Si), oxygen (O), and iron (Fe), with all evenly dispersed, confirming complete coating of the HGM by Fe_3_O_4_.

**Figure 2:**
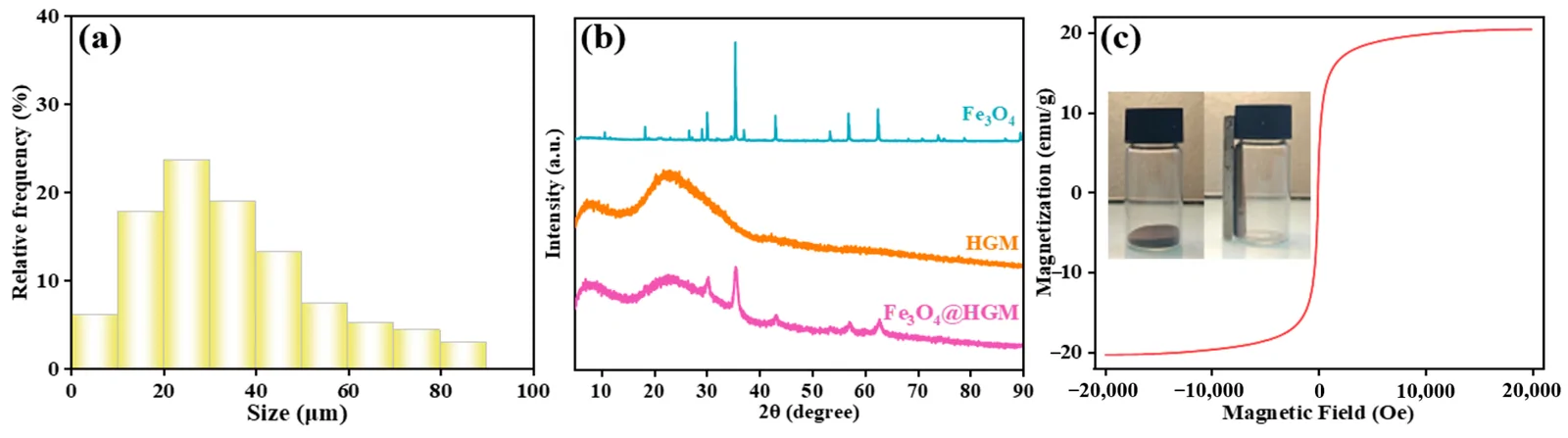
Physical properties of Fe_3_O_4_@HGM. (**a**) Particle size distribution of Fe_3_O_4_@HGM; (**b**) XRD patterns of HGM and Fe_3_O_4_@HGM; (**c**) magnetization curves of Fe_3_O_4_@HGM.

Figure 2a presents the particle size distribution (PSD) of Fe_3_O_4_@HGM, which follows a normal distribution with a mean particle size of 30.6 μm. X-ray diffraction (XRD) was employed to characterize the crystal structure and chemical composition (Figure 2b). Diffraction peaks are identified at 2θ = 30.2°, 35.5°, 43.2°, 53.6°, 57.2°, 62.7°, and 74.4°, which correspond to the (220), (311), (400), (422), (511), (440), and (533) crystal planes of Fe_3_O_4_, respectively [32]. Additionally, XRD analysis of the HGM raw material did not reveal any sharp characteristic peaks, but exhibited a hump-shaped peak near 2θ = 24°, indicating its amorphous nature accompanied by short-range crystalline structures—a hallmark characteristic of glassy materials [33].

Figure 2c shows the vibrating sample magnetometer (VSM) curve of Fe_3_O_4_@HGM. The saturation magnetization of the material is 20 emu/g, and the material can be readily drawn by an external magnetic field (inset of Figure 2c). The hysteresis loop shows no significant remanence or coercivity, indicating superparamagnetic behavior [34,35]. This property enables Fe_3_O_4_@HGM to respond rapidly to an applied magnetic field, enhancing the microwave absorption performance.

**Figure 3:**
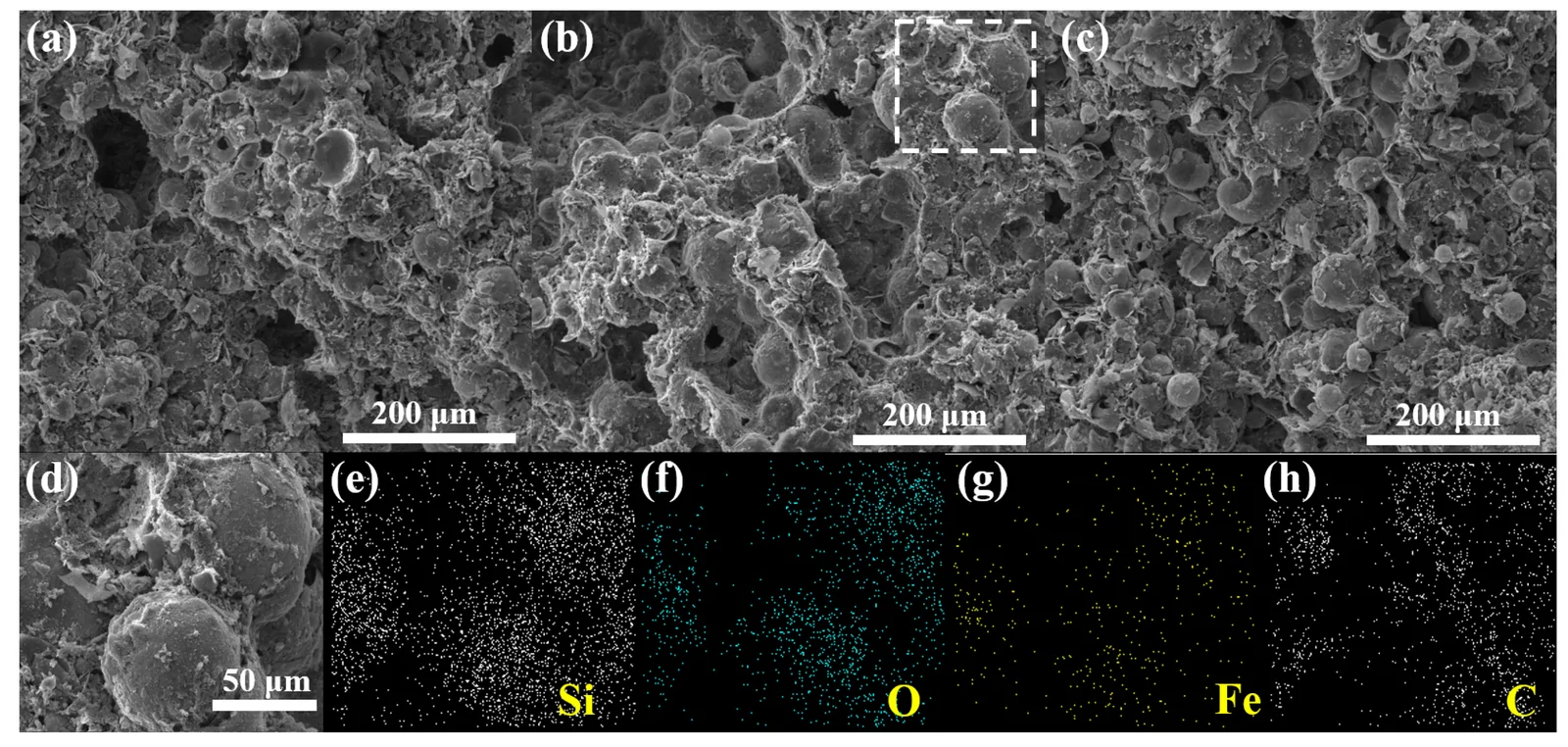
Morphology of G/Fe_3_O_4_@HGM composite foam with different Fe_3_O_4_@HGM content. SEM images of G/Fe_3_O_4_@HGM composite foam with (**a**) 25 wt%, (**b**) 35 wt%, and (**c**) 45 wt% Fe_3_O_4_@HGM; (**d**) enlarged view of the marked area in (**b**); (**e**–**h**) elemental distribution mapping of the area in (**d**).

Figure 3a–c shows SEM images of composite foam with different Fe_3_O_4_@HGM contents. Fe_3_O_4_@HGM is uniformly distributed, and its packing density increases with content. Figure 3d is a magnified image of the area highlighted in Figure 3b, showing firm bonding between Fe_3_O_4_@HGM and the coating matrix free of phase separation. Figure 3e–h shows elemental mapping: the spherical Fe_3_O_4_@HGM particles contain Si, O, and Fe, while the surrounding area is rich in carbon (C) from graphene and the organic group of silicone resin.

**Figure 4:**
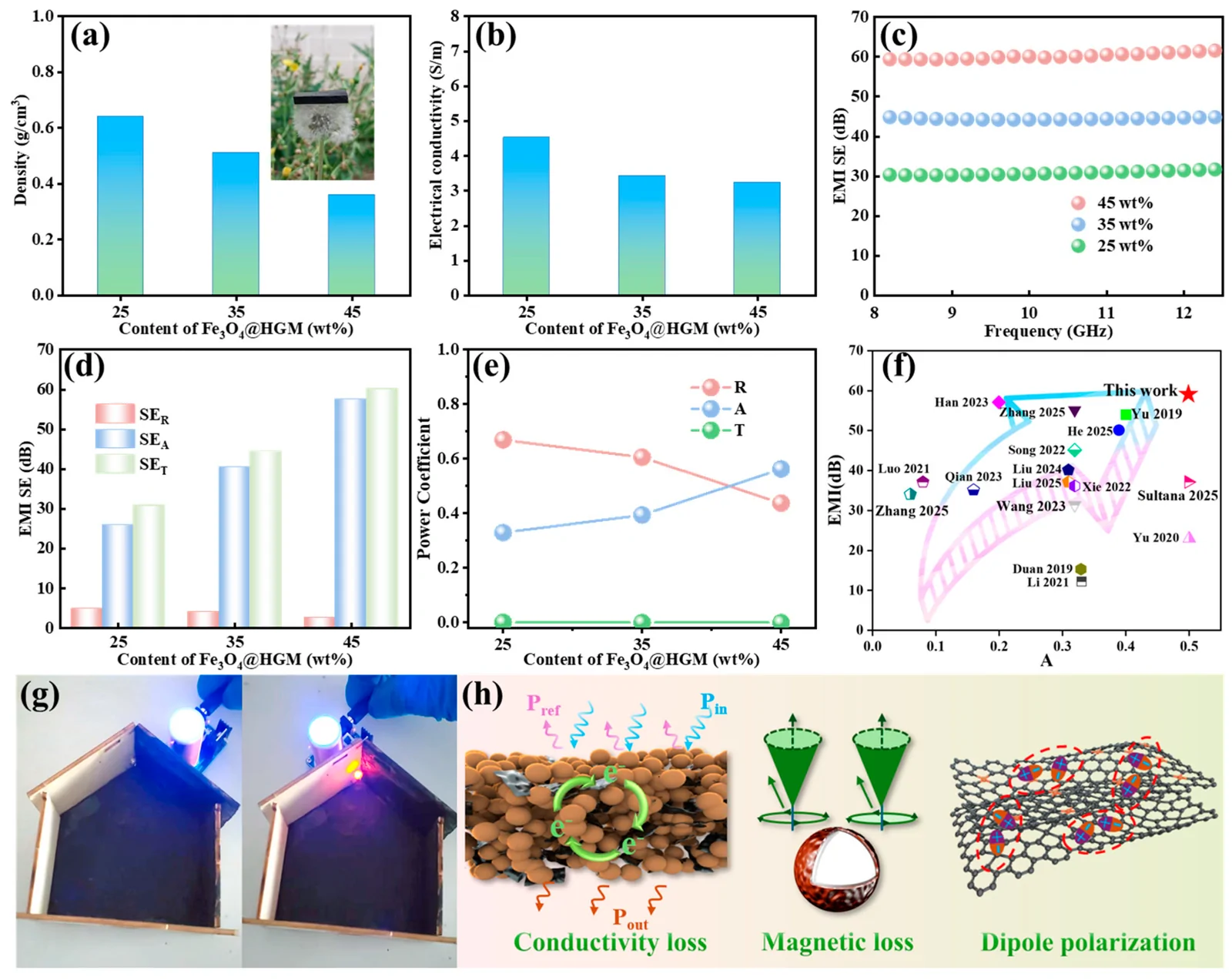
Conductivity and EMI shielding performances of G/Fe_3_O_4_@HGM composite foam. (**a**) Density, (**b**) conductivity, (**c**) EMI SE, (**d**) SER, SEA, SET, and (**e**) power coefficient of G/Fe_3_O_4_@HGM composite foam with different Fe_3_O_4_@HGM content; (**f**) comparison of reported EMI SE composites with this work [38,39,40,41,42,43,44,45,46,47,48,49,50,51,52]; (**g**) practical EMI shielding and (**h**) EMI shielding mechanism of G/Fe_3_O_4_@HGM composite foam.

Figure 4a shows the density variation in G/Fe_3_O_4_@HGM composite foam with Fe_3_O_4_@HGM content. With the increase in content, the density drops sharply, attaining a minimum value of 0.36 g/cm^3^. The foam is light enough to rest on a dandelion, demonstrating its ultralow density (inset of Figure 4a). Figure 4b shows the electrical conductivity of G/Fe_3_O_4_@HGM composite foam. Conductivity decreases with increasing Fe_3_O_4_@HGM content, due to the introduction of non-conductive hollow Fe_3_O_4_@HGM reducing the conductive pathways between graphene sheets.

Figure 4c presents the EMI shielding effectiveness (EMI SE) in the X-band (8.2–12.4 GHz). As the Fe_3_O_4_@HGM content rises from 25 wt% to 45 wt%, the average EMI SE grows from 30.7 dB to 60.1 dB. Figure S2 provides a comparison of G/HGM and G/Fe_3_O_4_@HGM foams: at a filler content of 45 wt%, the EMI SE values of these two foams are 38.4 dB and 60.1 dB, respectively. The superior EMI SE of the G/Fe_3_O_4_@HGM composite foam stems from the magnetic Fe_3_O_4_, which inherently possesses specific electrical conductivity, magnetic properties, and EMI shielding performance [36,37]. It synergizes with graphene to jointly endow the material with more excellent EMI shielding performance.

To investigate the shielding mechanism of the G/Fe_3_O_4_@HGM composite foam, Figure 4d presents its reflection shielding effectiveness (SER) and absorption shielding effectiveness (SEA). As Fe_3_O_4_@HGM content increases, this material’s SER value drops from 4.8 dB to 2.5 dB. This phenomenon could be attributed to two aspects: first, the introduction of magnetic nanoparticles enhances the material’s magnetic loss capability, which can effectively reduce EM wave reflection; second, the hollow microspheres provide channels for reciprocal reflection and absorption of electromagnetic waves, thereby amplifying the EM wave absorption capacity.

To further delineate the shielding mechanism of G/Fe_3_O_4_@HGM composite foam, Figure 4e displays the reflectivity (R), absorptivity (A), and transmissivity (T) data for the G/Fe_3_O_4_@HGM composite foam. The test findings show that as the doping content of Fe_3_O_4_@HGM filler increases, the absorption coefficient A of this composite rises, whereas the reflection coefficient R declines. For the 45 wt% Fe_3_O_4_@HGM composite foam, its average absorption coefficient A attains 0.56 (≥0.5), indicating the material employs an “absorption-dominant” shielding mechanism. For the composite foam incorporating 45 wt% HGM (Figure S2), the average absorption coefficient A of the fabricated G/HGM composite foam attains a value of 0.35, yet it is lower than that of the G/Fe_3_O_4_@HGM system with the same filler content. This outcome further validates that magnetic Fe_3_O_4_ plays a core role in elevating EM wave absorption and restraining secondary EM radiation contamination. At Fe_3_O_4_@HGM loadings of 25 wt% and 35 wt%, the average absorption coefficients A of G/Fe_3_O_4_@HGM composite foams attain 0.33 and 0.39, respectively. It can be concluded that the G/Fe_3_O_4_@HGM composite foam prepared in this work displays excellent EM wave absorption performance and is capable of efficiently restraining secondary EM radiation pollution.

Figure 4f contrasts the EMI shielding performance of the G/Fe_3_O_4_@HGM composite foam fabricated in this work with that of recently reported materials. The results demonstrate that compared with most existing studies [38,39,40,41,42,43,44,45,46,47,48,49,50,51,52], the G/Fe_3_O_4_@HGM composite foam not only possesses outstanding EMI SE but additionally has low reflectivity, displaying remarkable competitive advantages. Figure 4g shows a simulated house test model: part of the inner wall of the house is coated with a 1 mm thick composite foam containing 45 wt% Fe_3_O_4_@HGM. When the Tesla coil is placed on the side of the wall coated with the G/Fe_3_O_4_@HGM composite foam, the LED bulb inside the house remains off (Video S1), indicating that the EM waves generated by the Tesla coil cannot penetrate the wall coated with G/Fe_3_O_4_@HGM composite foam; when the Tesla coil is moved to the side of the wall without coating, the LED bulb inside the house lights up obviously, proving that the wall cannot effectively shield EM waves. This experiment further confirms the practical application value of G/Fe_3_O_4_@HGM composite foam in the role of an EMI shielding material. Figure 4h outlines the EMI shielding mechanism of G/Fe_3_O_4_@HGM composite foam. According to transmission line theory, when EM waves make contact with the shielding material, a segment of the EM waves undergoes reflection as a consequence of impedance mismatch at the air–foam interface, and the leftover portion penetrates the foam and is absorbed [53,54,55]. First, bare graphene exhibits a high dielectric constant without magnetic response, leading to severe impedance mismatch between the foam surface and air; most incident electromagnetic waves reflect immediately and cause secondary radiation pollution. After introducing Fe_3_O_4_@HGMs, magnetic Fe_3_O_4_ nanoparticles endow the composite with appropriate magnetic permeability, balancing the dielectric constant and magnetic permeability to optimize air–material impedance matching, allowing more electromagnetic waves to penetrate into the interior porous framework rather than reflecting at the surface. Second, multiple loss mechanisms synergistically dissipate microwave energy inside the foam: (1) conductive loss: interconnected graphene conductive networks convert electromagnetic energy into thermal energy via electron migration and collision; (2) magnetic loss: superparamagnetic Fe_3_O_4_ nanoparticles generate natural resonance, eddy current loss and hysteresis loss under alternating electromagnetic fields to consume magnetic field energy; and (3) interfacial polarization loss: abundant heterogeneous interfaces between the graphene–silicone matrix, HGM glass shell–Fe_3_O_4_ coating, and hollow pore–air phase produce space charge accumulation under electromagnetic excitation, triggering dipole polarization and interface polarization relaxation loss. Third, the interconnected porous structure and hollow microspheres construct multi-stage microwave-scattering cavities. Incident waves undergo repeated reflection–absorption–retransmission cycles inside pores and hollow spheres, extending transmission paths and fully activating the above three loss pathways. For the composite with 45 wt% Fe_3_O_4_@HGM, optimized impedance matching and maximized magnetic loss jointly increase the average absorption coefficient A to 0.56, realizing typical absorption-dominated shielding with an ultrahigh EMI SE of 60.1 dB.

**Figure 5:**
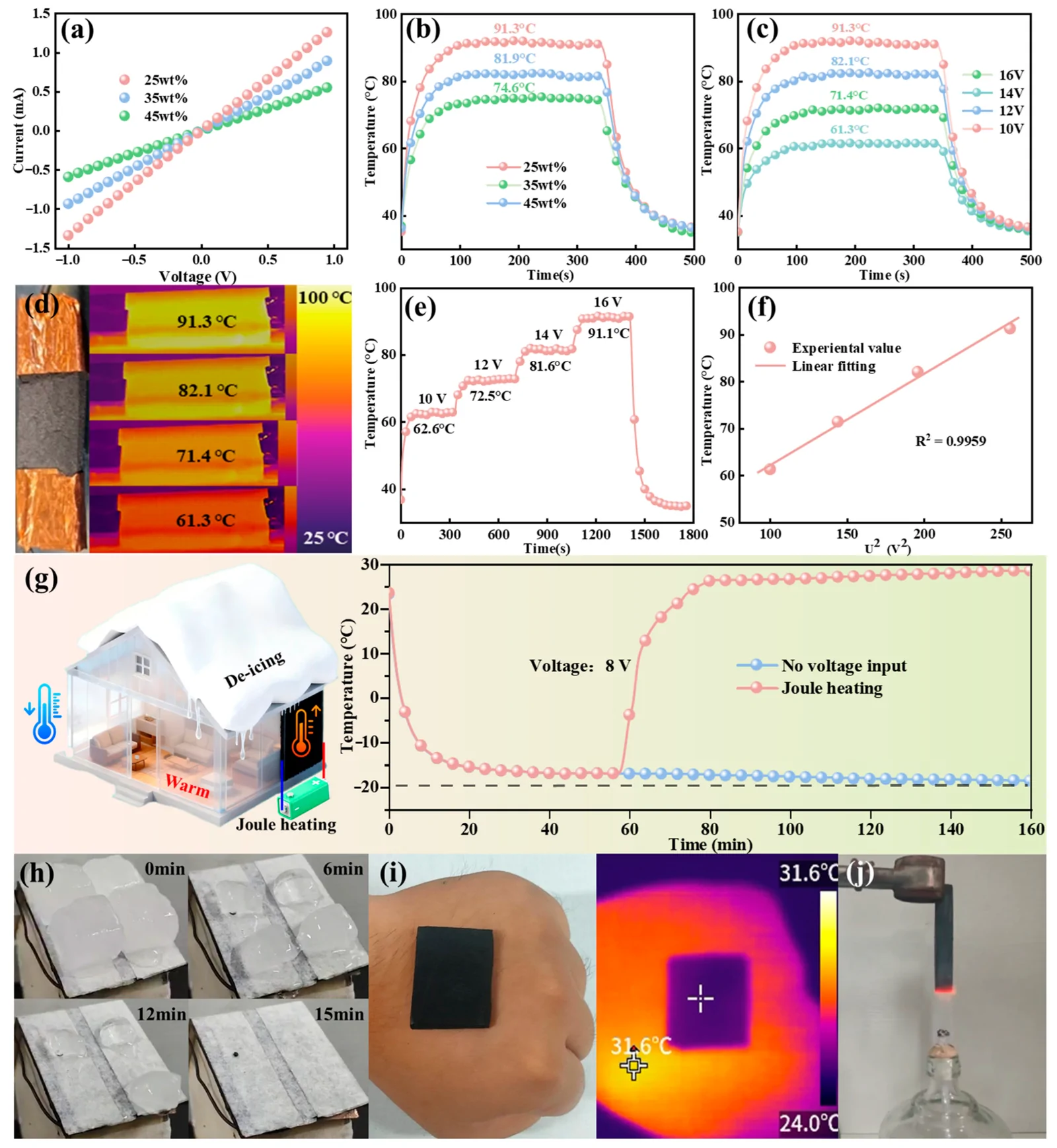
Thermal management capability of G/Fe_3_O_4_@HGM composite foam. (**a**) Current–voltage curves; (**b**) Joule heating performance at 16 V with different Fe_3_O_4_@HGM contents; (**c**) Joule heating performance of 25 wt% Fe_3_O_4_@HGM foam at different voltages and (**d**) corresponding IR images; (**e**) temperature variation under stepwise voltage regulation and (**f**) temperature vs. U^2^ fitting; (**g**) heating temperature curves and (**h**) de-icing test in a simulated house; (**i**) infrared stealth images and (**j**) flame-retardant test of G/Fe_3_O_4_@HGM composite foam.

In recent years, advanced EMI shielding materials, especially multifunctional composites integrating multiple performance requirements, have received extensive attention [56,57]. Given the excellent electrical conductivity of the G/Fe_3_O_4_@HGM composite foam, its multifunctional properties were further explored. For conductive polymer composites, the volt–ampere (I–V) characteristic is a core prerequisite for achieving stable Joule heating performance. Figure 5a shows the I–V curve of the G/Fe_3_O_4_@HGM composite foam, which presents a typical linear relationship, indicating that this material has application potential as a Joule heating material.

Figure 5b displays the Joule heating temperature curves of G/Fe_3_O_4_@HGM composite foams with different Fe_3_O_4_@HGM contents under 16 V voltage. As presented in the figure, the material’s heating temperature reduces alongside the rise in Fe_3_O_4_@HGM content. The primary cause of this phenomenon is that greater loading of Fe_3_O_4_@HGM tends to obstruct the conductive channels between graphene sheets, resulting in a reduction in the material’s electrical conductivity and consequently a decline in Joule heating efficiency. Under the same voltage of 16 V, when the contents of Fe_3_O_4_@HGM are 25 wt%, 35 wt%, and 45 wt%, the equilibrium temperatures of the material are 91.3 °C, 81.9 °C, and 74.6 °C, respectively.

Figure 5c,d shows the Joule heating temperature curve and infrared thermographic images of the G/Fe_3_O_4_@HGM composite foam with 25 wt% Fe_3_O_4_@HGM under different DC voltages, respectively. The results indicate that the material’s heating temperature increases substantially as the applied voltage rises: under 10 V, 12 V, 14 V, and 16 V, the stable heating temperatures of the foam material measure 61.3 °C, 71.4 °C, 82.1 °C, and 91.3 °C, respectively. The infrared thermographic images (Figure 5d) further confirm that the surface temperature of the material is uniformly distributed without local overheating, ensuring the stability of the heating performance.

Figure 5e depicts the temperature change curve of the G/Fe_3_O_4_@HGM composite foam during step-by-step continuous voltage adjustment via a variable transformer. These results show that the material’s temperature is capable of increasing rapidly and settling at the respective equilibrium temperature, demonstrating the material’s accessible temperature adjustability; furthermore, when the power supply is cut off, the material’s temperature can rapidly decrease to room temperature, demonstrating outstanding electrothermal responsiveness. More crucially, as illustrated in Figure 5f, the stable temperature of the foam has a linear positive correlation with the square of the applied voltage, with a correlation coefficient R^2^ of 0.9959. This demonstrates that the Joule heating temperature can be precisely adjusted by modifying the voltage, which is of substantial significance for practical applications. We further performed 20 continuous on/off cyclic stability tests at a fixed voltage of 10 V on the 25 wt% Fe_3_O_4_@HGM composite foam (Figure S4). The composite maintained nearly identical saturation equilibrium temperatures across all 20 heating-cooling cycles without obvious performance attenuation, which confirms the outstanding long-term electrothermal cycling stability of the conductive graphene network embedded in the silicone matrix. No structural cracking or conductive pathway damage was observed after cyclic testing, proving its reliability for long-duration de-icing and thermal management service.

Figure 5g presents the internal temperature variation curve of a simulated house model coated with 1 mm thick G/Fe_3_O_4_@HGM composite foam in a low-temperature environment (−20 °C). It can be seen from the figure that if the simulated house model is directly placed in a low-temperature environment of −20 °C, its internal temperature drops rapidly to −12.6 °C within 10 min and reaches thermal equilibrium with the external low-temperature environment after 20 min; however, when the composite foam is powered for Joule heating, the internal temperature of the simulated house rises swiftly to 10.8 °C within 3 min, reaches a level of 20.6 °C after 10 min of heating, and can be kept stably at 26.5 °C following 20 min of continuous heating. This result shows that the G/Fe_3_O_4_@HGM composite foam not only exhibits exceptional EMI shielding properties but is also suitable for use in building heating scenarios in winter. Figure 5h shows the physical image of ice cubes placed on the top of the simulated house, with the G/Fe_3_O_4_@HGM composite foam energized for Joule heating. As can be seen from the figure, the ice cubes start to melt rapidly after energization, and most of the ice cubes have melted after only 200 s. This indicates that the material can not only be used for building heating but also has good de-icing efficiency, expanding its application scenarios.

Figure 5i presents the infrared stealth performance of the G/Fe_3_O_4_@HGM composite foam. When the material covers the surface of a relatively warm object (such as the human body), the air in its internal microsphere structure can significantly reduce the overall thermal conductivity of the material, effectively inhibiting the heat transfer of the warm object to the outside and helping the material maintain a low surface temperature, thus showing excellent infrared stealth performance [58,59].

Figure 5i presents the infrared stealth performance of the G/Fe_3_O_4_@HGM composite foam. As observed from the figure, the fabricated G/Fe_3_O_4_@HGM composite foam exhibits no noticeable combustion or collapse during the test (Video S2), signifying its excellent fire resistance. Thus, this work provides a feasible approach for designing lightweight, multifunctional materials tailored for next-generation devices.

Although the G/Fe_3_O_4_@HGM foam exhibits excellent multifunctional performance, it has three practical drawbacks. First, its long-term electrothermal durability under humid and salt-fog conditions remains untested, as moisture may destroy graphene conductive networks. Second, poor surface wear resistance causes graphene shedding and conductivity loss under friction. Third, lab batch synthesis is unsuitable for low-cost, large-scale continuous production. Future work will address these issues: fabricate hydrophobic surface coatings for corrosion resistance; add flexible fillers to boost wear tolerance; optimize continuous low-temperature curing for scalable manufacturing; and develop flexible thin-film derivatives for wearable stealth and thermal management devices.

## 4. Conclusions

We successfully prepared lightweight G/Fe_3_O_4_@HGM composite foam with integrated EMI shielding, tunable Joule heating, infrared insulation and flame retardancy through simple mixing and ambient drying. The composite has an ultralow density of 0.36 g cm^−3^; the sample with 45 wt% Fe_3_O_4_@HGM achieves a maximum X-band EMI shielding efficiency of 60.1 dB and an average absorption coefficient of 0.56, realizing absorption-dominated shielding to reduce secondary electromagnetic pollution. At 16 V, the 25 wt% filler sample stabilizes at 91.3 °C, enabling rapid de-icing within 200 s at −20 °C and stable indoor heating, and the foam retains an intact structure under flame contact. We clarified the synergistic wave-absorbing mechanism combining graphene conductive loss, Fe_3_O_4_ magnetic loss and interfacial polarization, where optimized impedance matching dominates its excellent shielding capacity. To conclude, this work offers a robust strategy for designing high-performance, multifunctional composite materials. The graphene/Fe_3_O_4_@HGM foam exhibits considerable potential for use in advanced electronics, aerospace stealth technology, and intelligent heating systems.

## Data Availability

The original contributions presented in this study are included in the article/Supplementary Material. Further inquiries can be directed to the corresponding authors.

## Supplementary Materials

The following supporting information can be downloaded at https://www.mdpi.com/article/10.3390/molecules31162824/s1.

## References

[1] Liu A., Qiu H., Lu X.H., Guo H., Hu J.W., Liang C.B., He M.K., Yu Z., Zhang Y.L., Kong J. (2025). Asymmetric structural MXene/PBO aerogels for high-performance electromagnetic interference shielding with ultra-low reflection. Adv. Mater..

[2] Mao Y.S., Sheng Y.H., Fan Z.Y., Yang J., Liu J., Tang C.X., Fu S.H. (2025). Atmospheric pressure dried discontinuous pore gradient structured CNF-based aerogel for ultra-low reflection, broadband, and super-high EMI shielding. Adv. Funct. Mater..

[3] Zhang Z.C., Ding R., Su P.G., Zeng F.R., Jia X.X., Hu Z.Y., Wang Y.Z., Zhao H.B. (2025). Biomimetic ambient-pressure-dried aerogels with oriented microstructures for enhanced electromagnetic shielding. Adv. Funct. Mater..

[4] Yao L.H., Wang Y.C., Zhao J.G., Zhu Y.Q., Cao M.S. (2023). Multifunctional nanocrystalline-assembled porous hierarchical material and device for integrating microwave absorption, electromagnetic interference shielding, and energy storage. Small.

[5] Zhang Y.L., Liu A., Tian Y.Y., Tian Y.J., Qi X.S., Qiu H., He M.K., Zhou K., Gu J.W. (2025). Direct-ink-writing printed aerogels with dynamically reversible thermal management and tunable electromagnetic interference shielding. Adv. Mater..

[6] Liu H.G., Lei F.Y., Xu W.Y., Li Q.Q., Lei C., Xiong C.Y., Tian N., You C.Y., Yang Y. (2025). Designing carbon-foam composites via molten-state reduction for multifunctional electromagnetic interference shielding. ACS Nano.

[7] Nguyen V.T., Min B.K., Yi Y., Kim S.J., Choi C.G. (2020). MXene (Ti_3_C_2_T_X_)/graphene/PDMS composites for multifunctional broadband electromagnetic interference shielding skins. Chem. Eng. J..

[8] Kim J.H., Choi Y., Jang H.Y., Jiong S.H., Chen X.Q., Seo B.S., Choi W.J. (2024). Thermo-chemo-mechanically robust, multifunctional MXene/PVA/PAA-hanji textile with energy harvesting, EMI shielding, flame-retardant, and Joule heating capabilities. Adv. Mater..

[9] Li X.L., Li M.H., Li X., Fan X.M., Zhi C.Y. (2022). Low infrared emissivity and strong stealth of Ti-based MXenes. Research.

[10] Ge Y.F., Peng L., Pang Y.Q., Wang X.F., Cheng H.F., Liu D.Q. (2025). Spectrally selective thermal emitter for efficient infrared stealth compatible with microwave absorption. ACS Appl. Mater. Interfaces.

[11] Zhou Y.C., Yang J., Bai L., Bao R.Y., Yang M.B., Yang W. (2022). Flexible phase change hydrogels for mid-/low-temperature infrared stealth. Chem. Eng. J..

[12] Li M., Fang C., Cheng Y., Zhang X., Liu J., Xiang K., Zhang Y. (2025). Designed VO_2_/ANF/PVA aerogel composite material for adaptive infrared stealth and dynamic thermal regulation. Compos. Commun..

[13] Wang Y.J., Wang X.C., Kang M.M., Zhang Z.Z., Yang Y.C., Zeng W., Liu Z. (2025). Recent advances in graphene-based materials for radar and infrared stealth application. Compos. Part A Appl. Sci. Manuf..

[14] Mei J., Liao H.M., Yang S.Q., Liu M.T., Tu H., Wang J. (2025). Multiband electromagnetic interference shielding composite with dynamic thermal management and passive infrared stealth performance. ACS Appl. Mater. Interfaces.

[15] Wang S.J., Xiao X.L., Yan X., He W.W., Huang C.H., Chen L., Zhang H.X., Fang L., Yoon K.B. (2025). Multifunctional PDMS/Coal gasification fine slag composites for enhanced electromagnetic shielding and thermal management. Fuel.

[16] Wang Y.G., Zhong Y.Y., Kang J.H., Zhang B.L., Ma Z.L., Zhao Q.L., Li J.W. (2025). Multifunctional rigid polyimide foams with outstanding EMI shielding and wave absorption via densification strategy. J. Mater. Sci. Technol..

[17] Yan X., Yang J., Huang C., Yu Z., Zhang H.X., Fang L., Yoon K.B. (2025). Ultra-fast, cost-effective and scale-up preparation of lightweight electromagnetic interference shielding graphite worm-based aerogel. Chem. Eng. J..

[18] Meng L., Ma Y., Zou Y., Zhang B., Chen G., Dong C., Wang L., Guan H. (2024). Lightweight, breathable and self-cleaning polypyrrole-modified multifunctional cotton fabric for flexible electromagnetic interference shielding. Int. J. Biol. Macromol..

[19] Yan X., Zhang H.X., Gu W.H., Yoon K.B. (2025). Construction of self-healing polycaprolactone composite foams with segregated structure for superior electromagnetic interference shielding. J. Mater. Sci. Technol..

[20] Wang Z., Wang S., Du Z., Yang L., Cheng X., Wang H. (2023). Multifunctional wearable electronic textile based on fabric modified by MXene/AgNWs for pressure sensing, EMI and personal thermal management. Compos. Part B Eng..

[21] Eswaraiah V., Sankaranarayanan V., Ramaprabhu S. (2011). Functionalized Graphene–PVDF Foam Composites for EMI Shielding. Macromol. Mater. Eng..

[22] Fan B., Xing L., Yang K., Yang Y., Zhou F., Tong G., Wu W. (2023). Salt-templated graphene nanosheet foams filled in silicon rubber toward prominent EMI shielding effectiveness and high thermal conductivity. Carbon.

[23] Kang J.S., Zhang H.X., Yoon K.B. (2024). Hybrid rGO-CNT/epoxy nanocomposites as electromagnetic interference shielding materials. ACS Appl. Nano Mater..

[24] Wei Q., Li L., Deng Z., Wan G., Zhang Y., Du C., Su Y., Wang G. (2023). Scalable fabrication of nacre-structured graphene/polytetrafluoroethylene films for outstanding EMI shielding under extreme environment. Small.

[25] Cao M.S., Wang X.X., Cao W.Q., Yuan J. (2015). Ultrathin graphene: Electrical properties and highly efficient electromagnetic interference shielding. J. Mater. Chem. C.

[26] Hamidinejad M., Salari M., Ma L., Moghimian N., Zhao B., Taylor H.K., Filleter T., Park C.B. (2022). Electrically and thermally graded microcellular polymer/graphene nanoplatelet composite foams and their EMI shielding properties. Carbon.

[27] Li P., Zhang X., Liu Y. (2026). Recent research progress on graphene-based electromagnetic shielding materials. Carbon.

[28] Chithra A., Wilson P., Vijayan S., Rajeev R., Prabhakaran K. (2021). Thermally insulating robust carbon composite foams with high EMI shielding from natural cotton. J. Mater. Sci. Technol..

[29] He W., Wang S., Zhang H., Fang L., Wan Y. (2025). Lightweight multifunctional poly (dimethylsiloxane)/hollow microsphere/carbon nanotube composite foam for electromagnetic interference shielding and thermal management. ACS Appl. Polym. Mater..

[30] Kim E., Zhang H., Lee J.H., Chen H., Zhang H., Javed M.H., Shen X., Kim J.K. (2021). MXene/polyurethane auxetic composite foam for electromagnetic interference shielding and impact attenuation. Compos. Part A Appl. Sci..

[31] Wei Y., Bhuyan P., Kwon S.J., Kim S., Bae Y., Singh M., Thanh Tran D., Ha M., Jeong K.U., Ma X. (2024). Liquid metal grid patterned thin film devices toward absorption-dominant and strain-tunable electromagnetic interference shielding. Nano-Micro Lett..

[32] Huang C.H., Yan X., Wang S.J., Zhang H.X., Fang L., Yoon K.B. (2025). Preparation of self-healing thermoplastic polyurethane/carbon nanotube nanocomposites for electromagnetic shielding. Polym. Compos..

[33] Dalai S., Vijayalakshmi S., Shrivastava P., Sivam S.P., Sharma P. (2014). Preparation and characterization of hollow glass microspheres (HGMs) for hydrogen storage using urea as a blowing agent. Microelectron. Eng..

[34] Fang H., Guo H., Hu Y., Ren Y., Hsu P.C., Bai S.L. (2020). In-situ grown hollow Fe_3_O_4_ onto graphene foam nanocomposites with high EMI shielding effectiveness and thermal conductivity. Compos. Sci. Technol..

[35] Li X., Sheng M., Gong S., Wu H., Chen X., Lu X., Qu J. (2022). Flexible and multifunctional phase change composites featuring high-efficiency electromagnetic interference shielding and thermal management for use in electronic devices. Chem. Eng. J..

[36] Govindasamy T., Mathew N.K., Asapu V.K., Subramanian V., Subramanian B. (2024). Modulating the structural and magnetic properties of Fe_3_O_4_ NPs for high-performance supercapattery and EMI shielding applications. J. Energy Storage.

[37] Zhang S., Li W., Wu H., Jiao J. (2023). Multi-optimized flexible graphene oxide/multi-walled carbon nanotubes/ferroferric oxide nanopaper with enhanced electromagnetic wave absorption performance. Adv. Compos. Hybrid Mater..

[38] Han L., Li K., Liu H., Jiao Y., Yin X., Li H., Song Q., Qi L. (2023). Heterogeneous stacking strategy towards carbon aerogel for thermal management and electromagnetic interference shielding. Chem. Eng. J..

[39] Yu K., Zeng Y., Wang G., Luo X., Li T., Zhao J., Qian K., Park C.B. (2019). rGO/Fe_3_O_4_ hybrid induced ultra-efficient EMI shielding performance of phenolic-based carbon foam. RSC Adv..

[40] Sultana S., Shaik A.H., Rahaman A., Rahaman M., Chandan M.R. (2025). Recent advances in synthesis and processing of nanomaterial-based polymeric foams for EMI shielding applications. J. Mater. Sci..

[41] Wang H.R., Chen K.X., Shi Y.Q., Zhu Y.J., Jiang S.Q., Liu Y., Wu S.J., Fu L.B., Feng Y.Z., Song P.G. (2023). Flame retardant and multifunctional BC/MXene/MSiCnw/FRTPU aerogel composites with superior electromagnetic interference shielding via “consolidating” method. Chem. Eng. J..

[42] Qian H., Jiang W., Cao Q., Jiang B., Huang Y. (2023). Pushing electromagnetic interference shielding self-enhanced based on smart Ti_3_C_2_T_x_-WVO_2_ thermal management composite. Carbon.

[43] Liu T., Jiang W., Qian H., Shi X., Chen J., Cao Q., Li N., Huang Y., Jiang B. (2024). Nitrogen-coordinated borate esters induced entangled heterointerface toward multifunctional silicone/phenolic binary aerogel composite. Chem. Eng. J..

[44] Xie Y., Tang J., Ye F., Liu P. (2020). Microwave-assisted sintering to rapidly construct a segregated structure in low-melt-viscosity poly(lactic acid) for electromagnetic interference shielding. ACS Omega.

[45] Duan H., Zhu H., Yang J., Gao J., Yang Y., Xu L., Zhao G., Liu Y. (2019). Effect of carbon nanofiller dimension on synergistic EMI shielding network of epoxy/metal conductive foams. Compos. Part A Appl. Sci. Manuf..

[46] Zheng H., Pei X., Huang P., Zheng J., Guo G., Shen B., Wu F., Liu X., Zheng W. (2025). Elastomer foams with switchable folded cellular structure for EMI shielding, comprehensive sealing, and cushioning protection. Chem. Eng. J..

[47] He W., Li S., Wang S., Zhang H., Liu M., Yoon K.B. (2025). Facile fabrication of ultralight magnetic graphene/hollow microsphere aerogels for high performance shielding of electromagnetic wave, heat, and sound. Adv. Funct. Mater..

[48] Song P., Ma Z., Qiu H., Ru Y., Gu J. (2022). High-efficiency electromagnetic interference shielding of rGO@FeNi/epoxy composites with regular honeycomb structures. Nano-Micro Lett..

[49] Luo H., Lu Y., Qiu J. (2021). Double negative electromagnetic behavior and electromagnetic shielding performance of sandwich-like buckypaper/yttrium iron garnet-graphene aerogel/buckypaper metacomposites. Carbon.

[50] Liu H., Shao Y., Wang Z., Jiang L., Mou B., Tian N., You C., Li Y. (2024). Mechanically robust and multifunctional Ti_3_C_2_T_x_ MXene composite aerogel for broadband EMI shielding. Carbon.

[51] Li R., Ding L., Gao Q., Zhang H., Zeng D., Zhao B., Fan B., Zhang R. (2021). Tuning of anisotropic electrical conductivity and enhancement of EMI shielding of polymer composite foam via CO_2_-assisted delamination and orientation of MXene. Chem. Eng. J..

[52] Yu Z., Dai T., Yuan S., Zou H., Liu P. (2020). Electromagnetic interference shielding performance of anisotropic polyimide/graphene composite aerogels. ACS Appl. Mater. Interfaces.

[53] Sheng A., Ren W., Yang Y., Yan D.X., Duan H., Zhao G., Liu Y., Li Z.M. (2020). Multilayer WPU conductive composites with controllable electro-magnetic gradient for absorption-dominated electromagnetic interference shielding. Compos. Part A Appl. Sci. Manuf..

[54] Zheng X., Zhang H., Jiang R., Liu Z., Zhu S., Li W., Jiang X., Zhou X. (2023). Lightweight polyurethane composite foam for electromagnetic interference shielding with high absorption characteristic. J. Colloid Interface Sci..

[55] Sun B., Sun S., Guo Y., Mi H.Y., Jing X., Jiang X., Dong B., Liu C., Shen C. (2023). Asymmetric magnetic-electric dual-functional composite foams for ultra-efficient electromagnetic interference shielding with unprecedented low reflection. Compos. Part A Appl. Sci. Manuf..

[56] Tian K., Hu D., Wei Q., Fu Q., Deng H. (2023). Recent progress on multifunctional electromagnetic interference shielding polymer composites. J. Mater. Sci. Technol..

[57] Huang D., Chen Y., Zhang L., Sheng X. (2023). Flexible thermoregulatory microcapsule/polyurethane-MXene composite films with multiple thermal management functionalities and excellent EMI shielding performance. J. Mater. Sci. Technol..

[58] Ma Z., Jiang R., Jing J., Kang S., Ma L., Zhang K., Li J., Zhang Y., Qin J., Yun S. (2024). Lightweight dual-functional segregated nanocomposite foams for integrated infrared stealth and absorption-dominant electromagnetic interference shielding. Nano-Micro Lett..

[59] Zhang Z., Wang Z., Zhang Y., Zou P., Zhu M., Li S., Tang X., Lu Y., Li W., Lao K. (2025). Rigid polymethacrylimide composites foam for efficient electromagnetic dissipation and infrared stealth. Chem. Eng. J..

