# Supplementary material for: Construction of Graphene/Fe3O4@Hollow Glass Microsphere Composite Foam with Excellent Electromagnetic Interference Shielding, Joule Heating, and Flame-Retardant Properties
Source: Molecules. 2026 Aug 13;31(16):2824. doi: 10.3390/molecules31162824 (PMC13515506; doi:10.3390/molecules31162824)
Supplement: Supplementary file 1 [file molecules-31-02824-s001.zip › molecules-4469779-supplementary.pdf]

Supporting Information

Construction of Graphene/Fe_3O_4@Hollow Glass Microsphere Composite Foam with Excellent Electromagnetic Interference Shielding, Joule Heating, and Flame-Retardant Properties

Huan Yue [1,*], Shigang Li [2], Yixian Lv [1], Xueqing Wang [1], Jinlong Pan [1], Hao Wu [1], Heng Zhang [1] and Hexin Zhang [2,*]

[1] School of Chemistry and Chemical Engineering, Pingdingshan University, Pingdingshan 467000, China; 231005064@e.pdsu.edu.cn (Y.L.); 241005026@e.pdsu.edu.cn (X.W.); 251005009@e.pdsu.edu.cn (J.P.); 221027147@e.pdsu.edu.cn (H.W.); 241017069@e.pdsu.edu.cn (H.Z.)

[2] School of Chemistry and Chemical Engineering, Anhui University of Technology, Ma'anshan 243032, China; 2002421lsg@ahut.edu.cn

* Correspondence: 27141@pdsu.edu.cn (H.Y.); hxzhang@ahut.edu.cn (H.Z.)

Characterization

The morphology of HGM, Fe_3O_4@HGM and G/Fe_3O_4@HGM composite foams were examined using scanning electron microscopy (SEM; Nova NanoSEM 450). The crystalline structure of Fe_3O_4, HGM and Fe_3O_4@HGM was examined by X-ray diffraction (XRD, SmartLab 9 kW system, RIGAKU, Japan) with Cu Kα radiation and 45 kV. Magnetic properties of Fe_3O_4@HGM were characterized by a vibrating sample magnetometer (VSM, Lakeshore7404) at 300 K. The EMI SE of the G/Fe_3O_4@HGM composite foams were determined by measuring electromagnetic parameters in the frequency range of X-band (8-12 GHz) using a vector network analyzer (VNA, E8364B, Agilent, USA). Because the EMI SE tends to increase with the thickness of the specimen, then the thickness of the EMI measurement specimen was set at 1.0, 2.0 and 3.0 mm.

A certain voltage was supplied by a DC power supply (TPS605, 0~60V/0~5A, WANPTEK, China) to evaluate Joule heating performance. The conductive cupper tape was tightly applied to both ends of the G/Fe_3O_4@HGM composite foam to avoid contact resistance between the fixture and the sample. Thermal images of the samples

surface were collected by an infrared thermal imager equipment (PC200, Guide Sensmart, China) during Joule heating process. The electrical conductivity of the samples was measured with a DC resistance tester (TH2515, Tonghui Electron, China). The voltametric current signals were recorded using an electrochemical workstation (CHI660E, Shanghai Chenhua Device Company, China). The thermal conductivity of the material was measured by a thermal conductivity tester (TC3000E, Xiaxi, China).

The evaluation of the simulated house model Joule heating test employed a 3 mm-thick wooden substrate coated internally with approximately 1 mm of G/Fe_3O_4@HGM composite foam. Conductive copper tape was affixed internally as electrodes, spaced 2.7 cm apart, covering approximately 13.7 % of the room's total area. A schematic diagram of the internal structure of the simulated house model and its Joule heating test setup under low-temperature conditions is shown in Figure S1.

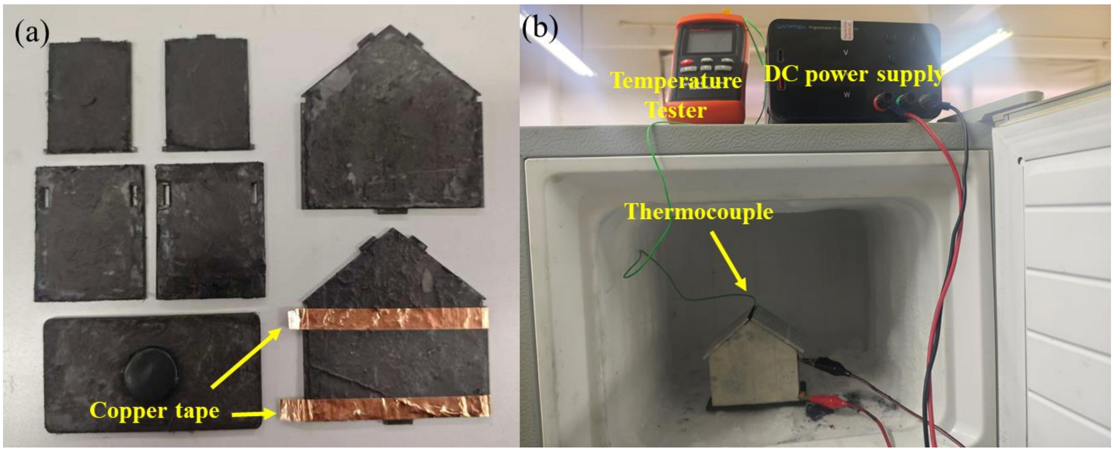

Figure S1. Schematic diagram of the (a) internal structure of the simulated house model and its (b) Joule heating test setup under low-temperature condition

Calculation of EMI SE

Scattering parameters (S_{11}, S_{12}, S_{22}, and S_{21}) were output by vector network analyzer directly. The power coefficients, reflection coefficients (R), transmission coefficients (T) and absorption coefficients (A), could be evaluated from the S parameters by following formulations:

$$R = |S_{11}|^2 = |S_{22}|^2 \qquad \text{(S1)}$$

$$T = |S_{12}|^2 = |S_{21}|^2 \qquad \text{(S2)}$$

$$A = 1 - R - T \qquad \text{(S3)}$$

EMI SE contained Total (SE_T), reflection (SE_R), absorption (SE_A) could be expressed as following formulations:

$$SE_T = -10\log(T) \qquad \text{(S4)}$$

$$SE_R = -10\log(1-R) \qquad \text{(S5)}$$

$$SE_A = -10\log(T/(1-R)) \qquad \text{(S6)}$$

EMI shielding efficiency is calculated according to below equation:

$$\text{Shielding efficiency (\%)} = 100 - \frac{1}{10^{\frac{SET}{10}}} \qquad \text{(S7)}$$

Joule's Law

$$Q = \frac{U^2}{R}t \qquad \text{(S8)}$$

Where Q is generated heat in joule, U is applied DC voltage, R is resistance and t is operating time.

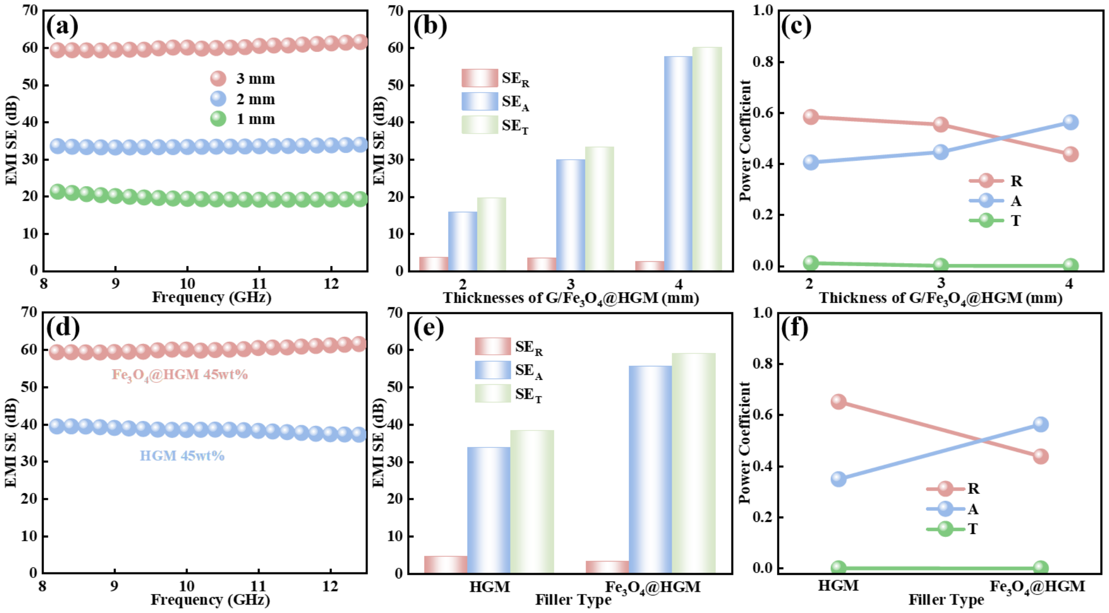

Figure S2. EMI shielding properties. (a) EMI SE, (b) SER, SEA, SET and (c) power coefficient of G/Fe3O4@HGM composite foam with 45 wt% Fe3O4@HGM; Comparison of (a) EMI SE, (b) SE$_R$, SE$_A$, SE$_T$ and (c) power coefficient of composite foam containing 45 wt% HGM or Fe3O4@HGM.

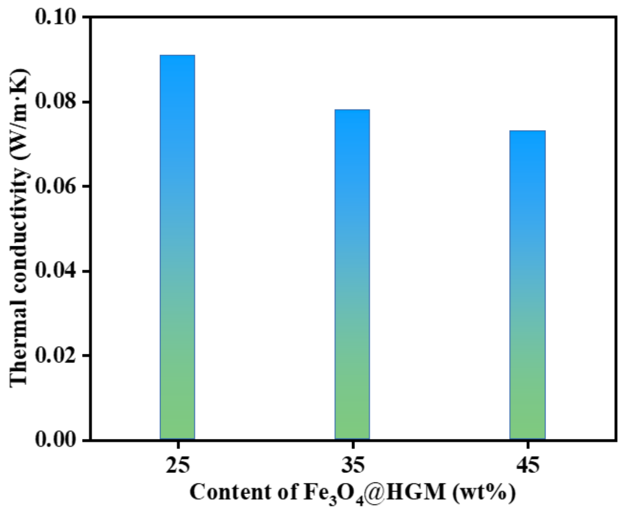

Figure S3. Thermal conductivity of G/Fe_3O_4@HGM composite foam with different Fe_3O_4@HGM content

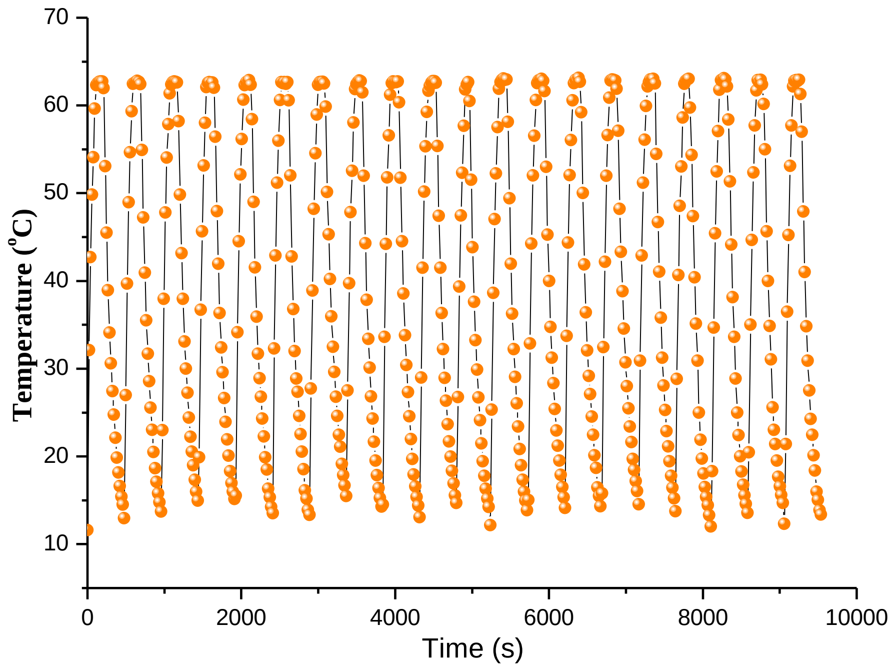

Figure S4. Continuous on/off cyclic stability tests at a fixed voltage of 10 V on the 25 wt% Fe_3O_4@HGM composite foam
